# The Role of *fosA* in Challenges with Fosfomycin Susceptibility Testing of Multispecies Klebsiella pneumoniae Carbapenemase-Producing Clinical Isolates

**DOI:** 10.1128/JCM.00634-19

**Published:** 2019-09-24

**Authors:** Zachary S. Elliott, Katie E. Barry, Heather L. Cox, Nicole Stoesser, Joanne Carroll, Kasi Vegesana, Shireen Kotay, Anna E. Sheppard, Alex Wailan, Derrick W. Crook, Hardik Parikh, Amy J. Mathers

**Affiliations:** a Department of Pharmacy Services, University of Virginia Health System, Charlottesville, Virginia, USA; b Division of Infectious Diseases and International Health, Department of Medicine, University of Virginia Health System, Charlottesville, Virginia, USA; c Modernizing Medical Microbiology Consortium, Nuffield Department of Clinical Medicine, John Radcliffe Hospital, Oxford University, Oxford, United Kingdom; d NIHR Health Protection Research Unit in Healthcare Associated Infection and Antimicrobial Resistance, University of Oxford in partnership with Public Health England, Oxford, United Kingdom; e Clinical Microbiology, Department of Pathology, University of Virginia Health System, Charlottesville, Virginia, USA; f Health Information & Technology, University of Virginia Health System, Charlottesville, Virginia, USA; g School of Medicine Research Computing, University of Virginia, Charlottesville, Virginia, USA; h NIHR Oxford Biomedical Research Centre, Oxford, United Kingdom; Medical College of Wisconsin

**Keywords:** carbapenemase-producing *Enterobacteriaceae*, *Enterobacteriaceae*, KPC, agar dilution, *fosA*, fosfomycin, susceptibility testing, whole-genome sequence

## Abstract

With multidrug-resistant (MDR) *Enterobacterales* on the rise, a nontoxic antimicrobial agent with a unique mechanism of action such as fosfomycin seems attractive. However, establishing accurate fosfomycin susceptibility testing for non-Escherichia coli isolates in a clinical microbiology laboratory remains problematic. We evaluated fosfomycin susceptibility by multiple methods with 96 KPC-producing clinical isolates of multiple strains and species collected at a single center between 2008 and 2016. In addition, we assessed the presence of fosfomycin resistance genes from whole-genome sequencing (WGS) data using NCBI’s AMRFinder and custom HMM search. Susceptibility testing was performed using a glucose-6-phosphate-supplemented fosfomycin Etest and Kirby-Bauer disk diffusion (DD) assays, and the results were compared to those obtained by agar dilution. Clinical Laboratory and Standards Institute (CLSI) breakpoints for E. coli were applied for interpretation. Overall, 63% (60/96) of isolates were susceptible by Etest, 70% (67/96) by DD, and 88% (84/96) by agar dilution. *fosA* was detected in 80% (70/88) of previously sequenced isolates, with species-specific associations and alleles, and *fosA*-positive isolates were associated with higher MIC distributions. Disk potentiation testing was performed using sodium phosphonoformate to inhibit *fosA* and showed significant increases in the zone diameter of DD testing for isolates that were *fosA* positive compared to those that were *fosA* negative. The addition of sodium phosphonoformate (PPF) corrected 10/14 (71%) major errors in categorical agreement with agar dilution. Our results indicate that *fosA* influences the inaccuracy of susceptibility testing by methods readily available in a clinical laboratory compared to agar dilution. Further research is needed to determine the impact of *fosA* on clinical outcomes.

## Abstract (precis)

With multidrug-resistant (MDR) *Enterobacterales* on the rise, a nontoxic antimicrobial agent with a unique mechanism of action such as fosfomycin seems attractive. However, establishing accurate fosfomycin susceptibility testing for non-Escherichia coli isolates in a clinical microbiology laboratory remains problematic. We evaluated fosfomycin susceptibility by multiple methods with 96 KPC-producing clinical isolates of multiple strains and species collected at a single center between 2008 and 2016.

## INTRODUCTION

Antimicrobial resistance among Gram-negative organisms continues to increase and presents a serious threat to modern medicine, with carbapenemase-producing *Enterobacteriaceae* (CPE) considered one of the most pressing issues (1). The concern with CPE is largely due to a lack of remaining therapeutic options, especially oral agents (2). This has led to the reevaluation of older antimicrobials to combat infections due to multidrug-resistant (MDR) Gram-negative pathogens. Fosfomycin, which was originally discovered in 1969, has been shown to have *in vitro* activity against CPE (3). In the United States, the oral formulation is available for the treatment of uncomplicated urinary tract infections due to susceptible strains of Escherichia coli and Enterococcus faecalis. Outside the United States, the intravenous (i.v.) formulation is approved and available for the management of systemic infections (4). Zavante Pharmaceuticals received fast-track designation from the FDA in 2015 for i.v. fosfomycin and has completed phase III clinical trials for the U.S. market (5).

Fosfomycin is a bactericidal antibiotic that binds to the cysteine residue of UDP-*N*-acetylglucosamine enolpyruvyl transferase (MurA) and inhibits peptidoglycan biosynthesis (6). Fosfomycin has activity against a range of bacterial pathogens, including highly drug-resistant *Enterobacteriaceae* (6). Fosfomycin resistance in *Enterobacterales* has been primarily driven by mutations in the *glpT* and *uhpT* genes, preventing active transport of fosfomycin into the cell (7). These mutations are, however, thought to be associated with a fitness cost in E. coli and are thus unstable (8, 9). The other major mechanism of resistance is hydrolysis of the drug via diverse Fos enzymes: FosA (FosA2, FosA3, FosA4, FosA5, FosA6, and FosA7), FosB, and FosX are metalloenzymes, whereas FosC is a serine enzyme (10). FosA was originally discovered on a transposon, Tn*2921*, in a Serratia marcescens plasmid and catalyzes the addition of glutathione to fosfomycin, rendering the drug ineffective (11). Transmissible *fosA* is of most concern in *Enterobacterales*, and plasmid-mediated *fosA3* has been increasingly identified in E. coli in Europe (12). A recent evaluation of Fos enzymes in non-E. coli *Enterobacterales* demonstrated that different *fosA* variants are chromosomally located in a species-specific manner (11). Lastly, MurA target site alteration can also confer fosfomycin resistance. Amino acid substitutions in MurA, most notably Asp369Asn and Leu370lle, have been responsible for fosfomycin resistance (13).

Susceptibility testing of fosfomycin for non-E. coli *Enterobacterales* is difficult for clinical microbiology labs (14–16). Both the CLSI and EUCAST specifically recommend against the use of broth microdilution methods, which likely impacts the inaccuracies with most automated susceptibility testing platforms for E. coli or Klebsiella pneumoniae (17, 18). Agar dilution is considered the reference method and is endorsed by the EUCAST; however, it is difficult to execute routinely in a clinical microbiology laboratory. Kirby-Bauer disk diffusion (DD) and Etests are more attractive options, as they can be performed easily in a clinical laboratory, but colonies often grow within the zones of inhibition, making interpretation difficult (19). Attempting to change the zone cutoff to better align with agar dilution has not proved successful with non-E. coli *Enterobacterales* (15). With agar dilution as the only accurate method for non-E. coli *Enterobacterales*, we aimed to characterize some of the molecular mechanisms (by whole-genome sequencing [WGS] for a subset of isolates) that may be contributing to the inaccuracies with these diffusion methods, using a diverse set of clinical, carbapenemase-producing strains and agar dilution-based reference phenotyping.

## MATERIALS AND METHODS

Ninety-six retrospective samples of Klebsiella pneumoniae carbapenemase (KPC)-producing *Gammaproteobacteria* isolates were selected from those collected at the University of Virginia Health System since August 2008. Isolates were chosen to represent diverse species and strains for which Illumina sequence data were available. Species identification had been performed by matrix-assisted laser desorption ionization–time of flight mass spectrometry (MALDI-TOF) (Vitek-MS, Vitek-2; bioMérieux) and all isolates were *bla*_KPC_ PCR positive as previously described (20).

Etest was performed using glucose-6-phosphate (G6P)-supplemented fosfomycin Etest strips (bioMérieux) according to the manufacturer’s instructions. DD was performed with a 200-μg fosfomycin disk with 50 μg of G6P (Becton and Dickinson, Franklin Lakes, NJ) on Mueller-Hinton agar as per CLSI guidelines (21). Agar dilution was performed using G6P-supplemented (25 μg/ml) Mueller-Hinton agar with fosfomycin concentrations ranging from 0.5 to 1,024 μg/ml per CLSI methods (22). A 0.5 McFarland inoculum for each isolate was placed in triplicate on the agar, placed in an incubator at 37°C for 16 to 20 h, and then interpreted. Plates were prepared without isolates at each concentration to serve as the control.

Per the package insert for interpretation of Etest, the crossing point of the ellipse was used to identify the MIC where colonies within the zone of the ellipse were accounted for if 5 colonies were present within 3 mm of the strip within the zone (bioMérieux). DD diameters were measured as the shortest distance between 2 separate colonies (17, 21). For agar dilution, the median of the three interpreted MICs was recorded as the result. All fosfomycin susceptibilities were interpreted according to CLSI breakpoints for E. coli urinary isolates, as there are no breakpoints available for non-E. coli *Enterobacterales* (17).

With agar dilution as the reference method, essential agreement for Etest was defined as MIC variation within 1 dilution. Categorical agreement was defined as matching susceptible/intermediate/resistant interpretation criteria for the two respective tests, as per CLSI guidelines for E. coli urinary isolates. Falsely susceptible results were deemed to be very major errors and falsely resistant results to be major errors. All other disagreements were deemed minor errors. Chi-square and Fisher’s exact tests were used to compare rates of nonsusceptibility and categorical agreement. The Mann-Whitney test was utilized for statistical analysis of MIC distributions and DD zone diameter changes.

Disk potentiation testing with sodium phosphonoformate (PPF) was performed as described by Ito et al. to specifically evaluate the activity of *fosA* enzymes and the impact on susceptibility testing with the disk diffusion method (11). Cultures of each isolate were plated on Mueller-Hinton agar with 1 mg of a 50-mg/ml sodium phosphonoformate (Sigma-Aldrich) solution added to a 200-μg fosfomycin disk supplemented with 50 μg of G6P. The plates were incubated overnight at 37°C and the inhibition zone was recorded. These inhibition zones were then compared the DD inhibition zones of fosfomycin without supplementation of PPF. For each isolate tested, a blank disk with PPF was also placed on the agar plate to serve as a negative control.

Molecular mechanisms of resistance to fosfomycin were investigated in a subset of isolates previously whole-genome sequenced by Ilumina Sequencing (HiSeq 2000) as previously described (20). The quality-filtered short reads were *de novo* assembled using SPAdes v3.11 (23), and the contigs were screened for *fos* resistance genes using NCBI’s AMRFiner (identity ≥ 0.9; coverage ≥ 0.5) (24). For isolates where AMRFinder failed to detect *fos* genes, we screened the contigs using a custom HMM model built from distinct *fosA* protein sequences published by Ito et al., with an E value threshold of 1e−20 (11, 25).

## RESULTS

### Fosfomycin susceptibility across species.

**FIG 1:**
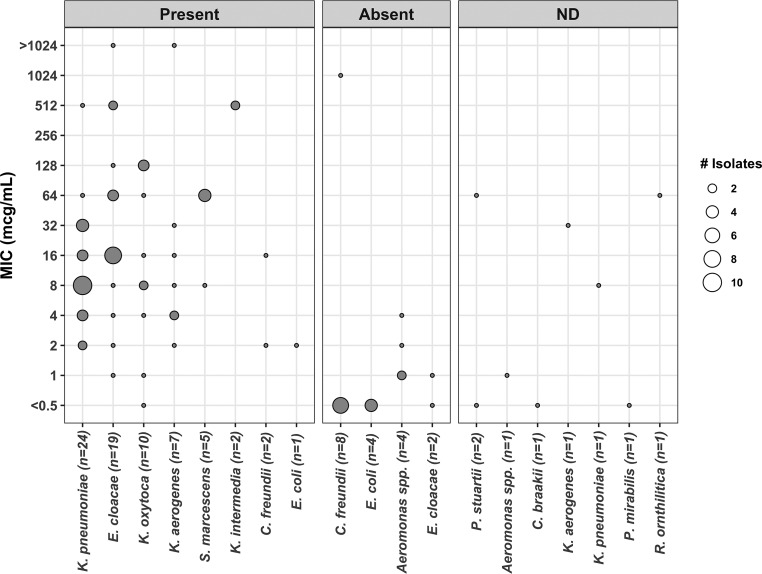
MIC distribution of KPC-producing isolates, grouped by *fosA* resistance gene presence screened from whole-genome sequencing data. ND, no sequencing data.

Ninety-six *bla*_KPC_-positive isolates across 12 species were included in the study (see Table 1 for species breakdown). Eighty-eight of the 96 isolates had undergone whole-genome sequencing (WGS). The MIC_50_ across all isolates was 8 μg/ml and the MIC_90_ was 128 μg/ml by agar dilution. Using the 2019 CLSI breakpoints (≤64 μg/ml indicates susceptibility), 84 of 96 isolates (88%) were susceptible, 11 of 96 (11%) were resistant, and 1 of 96 (1%) was classified as intermediate. The MIC distributions by agar dilution are shown in Fig. 1.

**TABLE 1:** Fosfomycin susceptibilities of isolates used in this study (*n* = 96)

Organism	No. (%) of isolates susceptible by the indicated test		
	Etest	DD	Agar dilution
Klebsiella pneumoniae (*n* = 25)	16 (64)	16 (64)	24 (96)
Enterobacter cloacae (*n* = 21)	10 (48)	15 (71)	17 (81)
*Citrobacter* spp. (*n* = 11)	10 (91)	9 (82)	10 (91)
Klebsiella oxytoca (*n* = 10)	7 (70)	8 (80)	7 (70)
Klebsiella aerogenes (*n* = 8)	3 (38)	3 (37.5)	7 (88)
Escherichia coli (*n* = 5)	5 (100)	5 (100)	5 (100)
Serratia marcescens (*n* = 5)	2 (40)	2 (40)	5 (100)
*Aeromonas* spp. (*n* = 5)	5 (100)	5 (100)	5 (100)
Klebsiella intermedia (*n* = 2)	0 (0)	0 (0)	0 (0)
Raoultella ornithinolytica (*n* = 1)	0 (0)	1 (100)	1 (100)
Providencia stuartii (*n* = 2)	1 (50)	1 (50)	2 (100)
Proteus mirabilis (*n* = 1)	1 (100)	0 (0)	1 (100)
Total	60 (63)	65 (68)	84 (88)

### Diffusion method performance.

**FIG 2:**
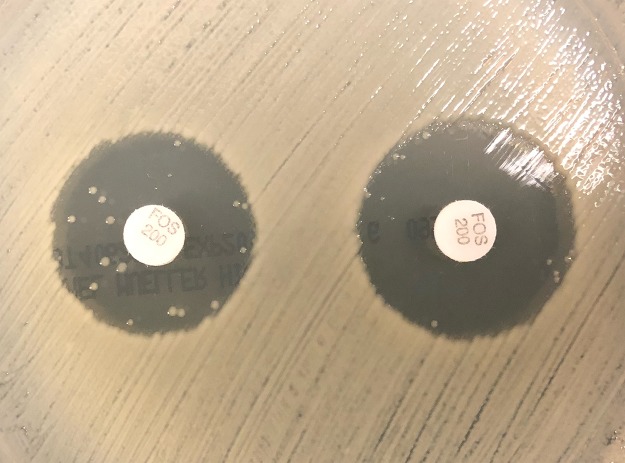
Example of elimination of fosfomycin-nonsusceptible subcolonies within zone of inhibition in *fosA6*-positive Klebsiella pneumoniae CAV 1217. Left, fosfomycin at 200 μg; right, fosfomycin at 200 μg plus PPF at 50 mg.

Sixty of 96 isolates (63%) were susceptible by Etest and 65/96 (68%) were susceptible by DD. Categorical agreement of Etest with agar dilution occurred in 69/96 isolates (72%), with 1 very major error, 16 major errors, and 10 minor errors. Essential agreement occurred in 55 of 96 isolates (57%) overall and in 4 of 5 (80%) E. coli isolates. Categorical agreement of DD with agar dilution occurred in 72/96 isolates (75%), with 2 very major errors, 14 major errors, and 8 minor errors. Of note, when testing the non-E. coli species, colonies within the zone were frequently present, making interpretation challenging but adhered to package insert and CLSI guidance for Etest and DD, respectively (21). Figure 2 shows a comparison of DD and Etest discordance in categorical agreement with agar dilution.

### *fosA* presence.

Of the isolates with WGS data, no isolate harbored *fosC*, while 70/88 isolates (80%) harbored an allele of *fosA*. All K. pneumoniae isolates (*n *=* *24) carried *fosA*, with 23 of 24 isolates (96%) harboring the *fosA6* or *fosA6*-like variant. Interestingly, only Klebsiella aerogenes isolates (*n* = 7; 100%) carried the same variant. Detection of *fosA* in all tested isolates is shown in Fig. 1. Isolates without *fosA* (*n *=* *18) had a MIC range of ≤0.5 to 1,024 μg/ml, a MIC_50_ of ≤0.5 μg/ml, and a MIC_90_ of 2 μg/ml. One of the 18 isolates (5.6%) was nonsuseptible to fosfomycin. Isolates harboring *fosA* (*n *=* *70) had a MIC range of ≤0.5 to >1,024 μg/ml, a MIC_50_ of 16 μg/ml, and a MIC_90_ of 128 μg/ml. Eleven of the 70 isolates (16%) were nonsusceptible to fosfomycin. Isolates carrying the *fosA* gene were associated with a higher MIC distribution than those without the gene (*P* ≤ 0.00001) but did not differ in rates of nonsusceptibility (*P = *0.26). These results are shown in Table 2.

**TABLE 2:** MICs of isolates with and without *fosA*

Isolate group	MIC (μg/ml)			*P* value	No. (%) of nonsuceptible isolates	*P* value
	Range	50%	90%			
Not harboring *fosA* (*n* = 18)	≤0.5 to 1,024	≤0.5	2	<0.00001	1 (5.56)	0.2627
Harboring *fosA* (*n* = 70)	≤0.5 to >1,024	16	128		11 (15.71)	

### FosA inhibition effect on susceptibility testing by diffusion method.

Disk potentiation testing with PPF was performed on all 96 isolates. Categorical agreement with agar dilution was found in 72/96 (75%) of isolates prior to the addition of PPF and in 81/96 (84%) isolates after the addition of PPF (*P* = 0.11). In the 88 isolates with WGS data available, rates of categorical agreement with and without PPF were compared. In isolates that were negative for the *fosA* gene, categorical agreement was found in 18/18 (100%) and 17/18 (94%) of isolates before and after the addition of PPF, respectively (*P* = 1). In isolates that carried the *fosA* gene, categorical agreement was found in 49/70 (70%) and 59/70 (84%) before and after the addition of PPF, respectively (*P* = 0.04). When specifically isolating all nonsusceptible isolates by DD, categorical agreement was found in 9/31 (29%) isolates and 20/31 (65%) before and after the addition of PPF, respectively (*P* = 0.005). Results are shown in Tables 3 and 4. The presence of PPF not only increased the zone size but also decreased the presence of colonies within the zone in DD testing for the *fosA*-positive isolates (Fig. 2). The control disk with PPF alone indicated no antibacterial activity, with no isolates demonstrating a zone of inhibition.

**TABLE 3:** Disk potentiation testing on all isolates (*n* = 96)

DD condition	Susceptibility category	No. of isolates	Categorical agreement, no./total (%)
Fosfomycin only	Susceptible	65	63/65 (96.9)
	Intermediate	7	0/7 (0)
	Resistant	24	9/24 (37.5)
Total			72/96 (75)
Fosfomycin + PPF	Susceptible	80	76/80 (95.0)
	Intermediate	6	0/6 (0)
	Resistant	10	5/10 (50)
Total			81/96 (84)
*P* value			0.10644
			

**TABLE 4:** Disk potentiation testing on WGS isolates (*n* = 88)

Organism group and DD condition	Susceptibility category	No. of isolates	Categorical agreement, no./total (%)	*P* value
FosA negative (*n* = 18)				
Fosfomycin only	Susceptible	17	17/17 (100)	
	Intermediate	0	NA^*a*^	
	Resistant	1	1/1 (100)	
Total			18/18 (100)	1
Fosfomycin + PPF	Susceptible	17	17/17 (100)	
	Intermediate	1	0/1 (0)	
	Resistant	0	NA	
Total			17/18 (94.44)	
				
FosA positive (*n* = 70)				
Fosfomycin only	Susceptible	43	41/43 (95.3)	
	Intermediate	6	0/6 (0)	
	Resistant	21	8/21 (38.1)	
Total			49/70 (70.0)	0.04415
Fosfomycin + PPF	Susceptible	58	54/58 (93.1)	
	Intermediate	4	0/4 (0)	
	Resistant	8	5/8 (62.5)	
Total			59/70 (84.3)	

a NA, not applicable.

## DISCUSSION

We demonstrate that fosfomycin susceptibility testing by routinely used laboratory diffusion-based methods (Etest and DD) largely overcalls resistance compared to agar dilution as a gold standard (Table 1). Fosfomycin susceptibility appears to be influenced by the presence of *fosA* among these KPC-producing *Enterobacterales* isolates. This is highly relevant to the clinical microbiologist who is frequently fielding requests for fosfomycin susceptibility testing for non-E. coli *Enterobacterales*. A prior study by Kaase et al. tested 107 carbapenem-nonsusceptible *Enterobacteriaceae* isolates, of which 80 produced various carbapenemases (KPC, VIM, NDM, and OXA-48), and they found 81% of isolates to be susceptible to fosfomycin, with a MIC of ≤64 μg/ml by agar dilution. This study also found similar issues of discordance with diffusion testing methods (15), as was also seen in a study by Hirsch et al. (14).

Fosfomycin has been promoted as a useful, safe medication for the treatment of urinary tract infections against multidrug-resistant non-E. coli *Enterobacteriaceae* (2, 3, 7, 26, 27), but susceptibility testing by agar dilution is practically difficult for a clinical microbiology lab. The CLSI cutoffs for E. coli were applied for Etest and DD interpretation for all species tested in this experiment, which requires accounting for scattered colonies within the ellipse per the package insert or zone per the CLSI (17). Fosfomycin susceptibility testing for E. coli was reviewed by the CLSI in 2018. At that time the recommendation that the susceptibility cutoffs apply only to E. coli was strengthened, and since scattered colonies are rare within the zone for this species, the practice of measuring the zone from the innermost colonies was upheld (17). This differs from the new EUCAST guidelines for fosfomycin and E. coli, which suggest ignoring scattered colonies within diffusion-based inhibition zones and utilizing agar dilution approaches for non-*E.coli Enterobacterales* (28). This decision for the former was based on the findings that subcolonies of E. coli within inhibition zones are rare (<1% of isolates) and largely less fit, with channel or transporter mutations (8, 9).

We postulate based on our findings that the colonies within the inhibition zone seen more frequently with some non-E. coli species may be driven by the presence of a chromosomal *fosA* gene rather than channel or transporter mutations. Thus, the advice to ignore the colonies in the zone in E. coli may not apply to non-E. coli *Enterobacterales*, and clinical microbiologists should proceed with caution. The discordance among commercially available DD and Etest with agar dilution we observed was largely due to bacterial colonies that grew within the zone of inhibition. Based on the change in zone size with the addition of a FosA inhibitor as well as the work of others demonstrating the activity of chromosomally expressed FosA, it may unwise to ignore subcolonies, as the clinical implications of this finding remain unknown (29).

In our subset of E. coli isolates, categorical agreement was found in 5 of 5 (100%) isolates. All E. coli isolates were susceptible to fosfomycin by both DD and agar dilution, and no colonies were observed within the zones of inhibition. Although our numbers are small, this is consistent with other reports (8).

*fosA* (alleles 1 to 7) was identified in the majority of the clinical isolates in this study. *FosA* was present in all *Klebsiella* species isolates, with a large portion harboring the *fosA6* allele. *fosA6* was first reported in 2016, from an extended-spectrum-β-lactamase-(ESBL)-producing, fosfomycin-resistant E. coli strain in Pennsylvania. It shared 96% identity with *fosA5* and 79% identity with *fosA3* but was located on a plasmid, unlike the chromosomally borne *fosA* in K. pneumoniae (30). It has been suggested that *fosA6* was mobilized from the chromosome of K. pneumoniae to an E. coli plasmid (30). However, in our study, no E. coli harbored *fosA6*, but rather one isolate harbored *fosA7*, which has been described to be on the chromosome of Salmonella enterica (31). We postulate that this gene may have been acquired via plasmid transfer, with Salmonella enterica serving as the reservoir for this allele. No *fosC* was detected, as expected, as it is a gene found most commonly within *Pseudomonas* spp., which were not included in this study.

In this subset of isolates, the presence of *fosA* resulted in a trend toward higher MIC values than for isolates not harboring the gene. Despite the higher distribution of MIC values, there was no statistical difference in fosfomycin susceptibility when determined by the agar dilution method. However, the E. coli breakpoints used in this study are based on obtainable urinary concentrations. The impact of increased MIC distributions on fosfomycin susceptibility in nonurinary sources of infection is unclear, as MIC breakpoints will likely be lower.

Disk potentiation testing with PPF was performed to specifically evaluate the activity of *fosA* enzymes and the impact on susceptibility testing with DD. The addition of PPF significantly increased zone diameter size and, subsequently, improved categorical agreement of disk diffusion with agar dilution, particularly in *fosA*-positive isolates, in which most major errors were eliminated. These improvements were largely due to the elimination of subcolonies within the zone of inhibition, as illustrated for isolate CAV 1217 (Fig. 2). As expected, there was no statistical change in categorical agreement in isolates that did not harbor a *fosA* allele. The results of the disk potentiation testing indicate that *fosA* impacts fosfomycin activity and limits convenient diffusion-based susceptibility testing. Some isolates had substantial zone diameter increases (6 to 8 mm) after the addition of PPF, yet this did not alter the susceptibility interpretation. This is likely due to alternative mechanisms of fosfomycin resistance, such as transporter mutations or MurA mutations. An alternative explanation is that certain alleles of *fosA* may have the ability to overcome the inhibition of PPF that was added to the disk.

Lastly, our data suggest that the addition of PPF may have a synergistic effect with fosfomycin against *fosA*-positive organisms. This is corroborated by a recently published study that found significant MIC reductions and restored fosfomycin susceptibility in *fosA*-positive Gram-negative organisms (29). PPF is available as the antiviral foscarnet but would likely be unattractive as an adjuvant therapy due to toxicity.

Our study is limited by a small sample size of *bla*_KPC_-positive multidrug-resistant isolates collected from a single center. We are unable to make inferences on non-KPC-positive isolates, as they were not included in this study. However, the clinical utility of fosfomycin is primarily against MDR isolates, and our study highlights the difficulty in accurately providing fosfomycin antimicrobial susceptibility testing (AST) for these organisms. A further limitation is the lack of exploration of other molecular mechanisms of resistance, which were not evaluated in all isolates. Our study also lacks outcome data, and therefore, we can make no conclusions on the clinical implications of fosfomycin susceptibility testing results.

In conclusion, fosfomycin appears to have reliable *in vitro* activity against KPC-producing Gram-negative organisms by agar dilution. However, methods readily available in a clinical microbiology laboratory, Etest and DD, generate frequent major errors, with the presence of *fosA* impacting the interpretation of these diffusion-based methods. Caution is advised when interpreting and releasing AST results derived from diffusion-based methods for non-E. coli *Enterobacterales*. Regardless, further research is needed to establish correlations between antimicrobial susceptibility testing, *fosA* presence, and clinical outcomes.
